# Age, gender, and setting's effect on community pharmacists' stress and confidence in the COVID-19 pandemic

**DOI:** 10.1016/j.rcsop.2023.100239

**Published:** 2023-03-09

**Authors:** Stephanie A. Gernant, Stefanie C. Nigro, Dean G. Cruess, Marie Smith, Nathaniel M. Rickles

**Affiliations:** a Department of Pharmacy Practice, University of Connecticut School of Pharmacy, 69 North Eagleville Rd. Storrs, CT 06229, United States of America; b Department of Psychological Sciences, University of Connecticut, 406 Babbidge Road, Storrs, CT 06269, United States of America

**Keywords:** Stress, Pharmacists, Community pharmacies, COVID-19 pandemic, Disaster medicine

## Abstract

**Background:**

Reports of increased stress among healthcare workers were commonplace during the early days of the COVID-19 pandemic, but little is known about community pharmacists' experiences.

**Objective:**

To characterize community pharmacists' stress and confidence during the early COVID-19 pandemic and identify associated factors.

**Methods:**

Pharmacists who worked in a brick-and-mortar community pharmacy (e.g., big-box, chain, independent, or grocery pharmacies) located in Connecticut and had regular face-to-face interaction with the public were surveyed. Survey items were selected from the Perceived Stress Scale-10 (PSS-10) and adapted from the Emergency Risk-Communication (ERC) framework. Data were analyzed using chi-square and ANOVA.

**Results:**

Survey results suggested pharmacists experienced moderate levels of stress, as negative responses to PSS-10 items ranged between 6.4% to 43.3%, respectively. Overall, pharmacists had high rates of confidence in their ability to manage the pandemic, agreeing or strongly agreeing that they could manage their own mental health (73.1%), and communicate the risks of the pandemic (72.0%). However, 28.0% reported that they had avoided talking about the pandemic because it made them feel “stressed, or nervous.” Women and those working in chain community pharmacies tended to report significantly higher rates of stress to several items in the PSS-10 compared to men and pharmacists working in non-chain settings. Women and chain community pharmacists were also significantly more likely to report overall that they had avoided talking about public health risks because it made them feel anxious, stressed, or depressed (29.4% men vs. 34.5% women χ^2^ (4) > 22.6, *p* < 0.01). However, confidence to communicate critical risk messages neither differed between men and women (77.6% men vs. 68.8% women χ^2^ (4) > 8.3, *p* = 0.08), nor between chain and non-chain community pharmacists (71.0% chain vs. 73.7% non-chain χ^2^ (4) > 8.9, *p* = 0.32).

**Conclusion:**

Being female, younger age, and employed at a chain pharmacy were associated with higher rates of stress and lower self-confidence among community pharmacists during the COVID-19 pandemic.

## 1 Background

Community pharmacy is a high-stress working environment due to its high-risk, fast-paced, and interruptive nature. These interruptions often occur because community pharmacists have long served as the most accessible healthcare professional in the United States (U.S.). The importance of pharmacists' accessibility was highlighted at the onset of the COVID-19 pandemic in early 2020 when public health officials implemented universal social distancing guidelines. As part of these guidelines, some industries like food distribution and healthcare were deemed ‘critical’ by the U.S. Department of Homeland Security (DHS) to the nation's public health, safety, and economic/national security. Community pharmacists were included as ‘essential critical infrastructure workers’ and were thus guided by DHS to maintain a normal work schedule.^1^ As such, community pharmacies remained open and accessible to the public when many primary and ambulatory clinics closed for social distancing.

Before the pandemic, rates of burnout and job dissatisfaction were high, as approximately one-out-of-five community pharmacists reported that work-related stress was so poor that it adversely affected their mental/physical health, quality of work, or personal relationships.^2^ An inherent physical risk of remaining open and accessible to the public during the pandemic, coupled with changes to workload, staffing, and social distancing likely compounded pharmacists' stress.^3^ Indeed, during the early months of the pandemic, reports of significantly increased stress among healthcare workers were commonplace, with the media recognizing stress and burnout among hospital staff as frontline workers.^4^ Literature on U.S. pharmacists' stress during the COVID pandemic is scarce, with only a few surveys done outside of the country at the time of this research.^3^^,^^5^^,^^6^ Much remains unknown about what community pharmacists faced as one of many other ‘forgotten’ groups of ‘essential frontline workers in the U.S.

### 1.1 Objective

This report aimed to characterize community pharmacists' stress and confidence during the early months of the COVID-19 pandemic and identify demographics associated with varying levels of that stress and confidence.

## 2 Methods

An online survey of community pharmacists (e.g., big-box, chain, independent, or grocery pharmacies) in the State of Connecticut was conducted between July 29–September 29, 2020.

Four-hundred respondents were sought for participation as a convenience sample. Respondents included pharmacists licensed in Connecticut who attested to (1) having practiced on average at least 20 hours a week after March 1, 2020, in a brick-and-mortar community pharmacy physically located in Connecticut; and (2) having regular and physical face-to-face interactions with the public in their provision of pharmacy services. No other requirements, such as time licensed or degree earned were required. Pharmacists working remotely, in medical marijuana dispensaries, or in other non-community pharmacy settings were excluded from the study. This study was reviewed and approved by the University of Connecticut Institutional Review Board. Participants' responses were collected anonymously.

Survey items that measured perceptions of confidence were developed from domains in the Emergency Risk Communication (ERC) framework.^7^ Developed in 2017 by Savoia, Lin, and Gamhewage, the ERC was chosen as this study's conceptual framework as it serves to guide the development of studies that assess communication outcomes related to public health emergencies. Domains of the ERC are broadly categorized into three groups, including (1) information environment, (2) populations, and (3) public health systems. Respective to each domain, three correlating items questioned respondents' confidence in (1) proving information to the public, (2) managing their own and others' mental health, and (3) performing point of care testing (POCT) mitigation. A fourth item sought to explore if stress (i.e., “risk perception, emotions and trust” in the ERC) related to pharmacists' avoidance of risk communication (i.e., “knowledge, attitudes and practices” in the ERC).

Respondents were also asked questions from the Perceived Stress Scale (PSS-10), developed by Cohen et al. in 1983.^8^ In the PSS-10, respondents were asked how often they feel a certain way on a five-point scale from ‘never’ (1) to ‘very often’ (5). To calculate a total PSS-10 score, responses to the positively stated items were reversed and then summed across all items. Higher scores indicate higher levels of perceived stress. Other items included a mix of multiple-choice and Likert scales; free-text boxes were included without forced response for the participant to provide comments or clarification.

Two community pharmacists matching the inclusion/exclusion criteria beta-tested the survey for clarity and content before distribution; their data were not included in the analysis.

### 2.1 Data collection

An anonymous e-survey was developed and distributed by email through listservs held by the <REDACTED> Continuing Education Department, The New England Pharmacy Community Research Network, and the Connecticut Pharmacists Association. Respondents accessed the e-survey via a Qualtrics® link and were compensated with a ten-dollar incentive as a token of appreciation for their time.

### 2.2 Data analysis

**Fig. 1:**
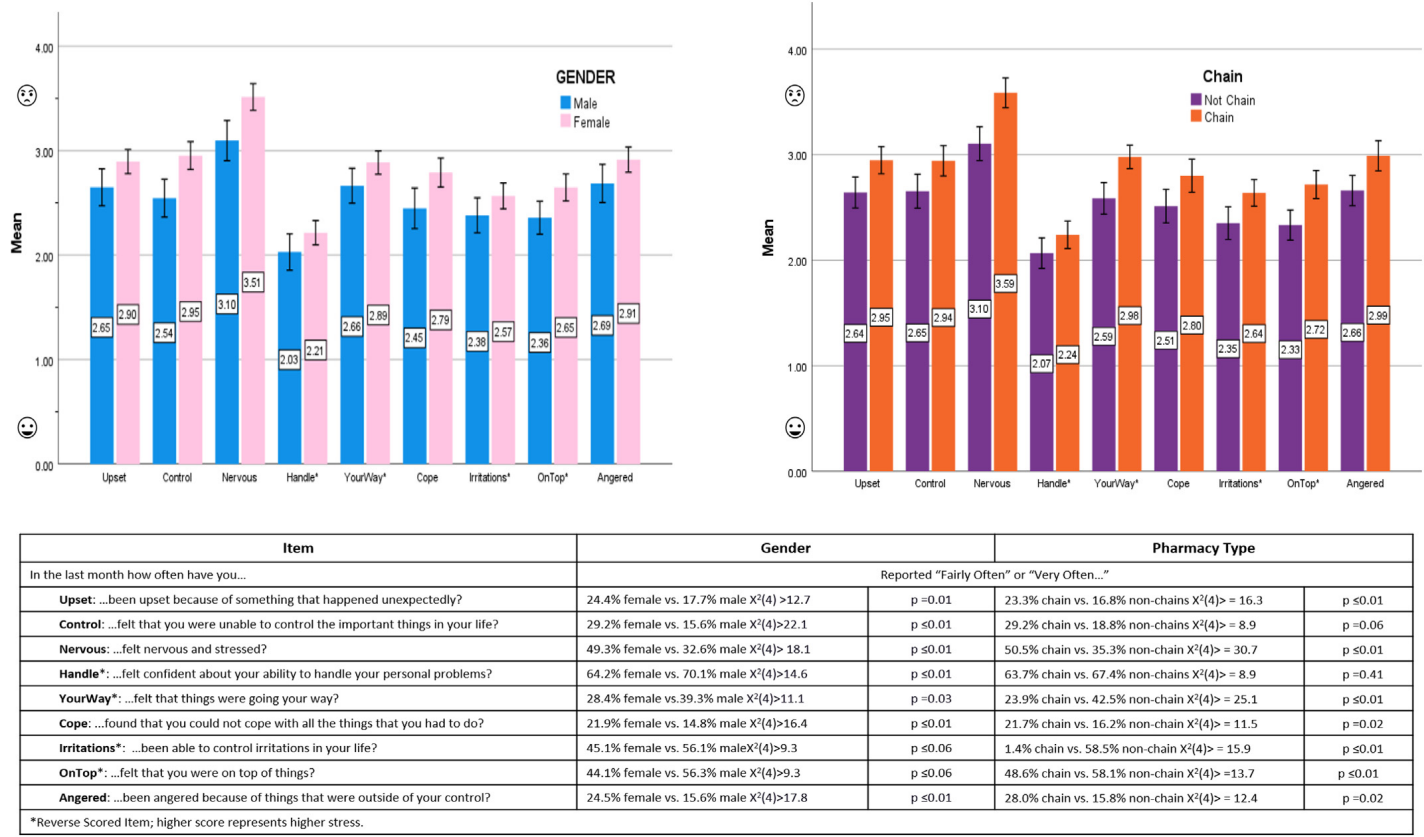
Community pharmacists' reported stress by gender and practice site during the COVID-19 pandemic.

**Fig. 2:**
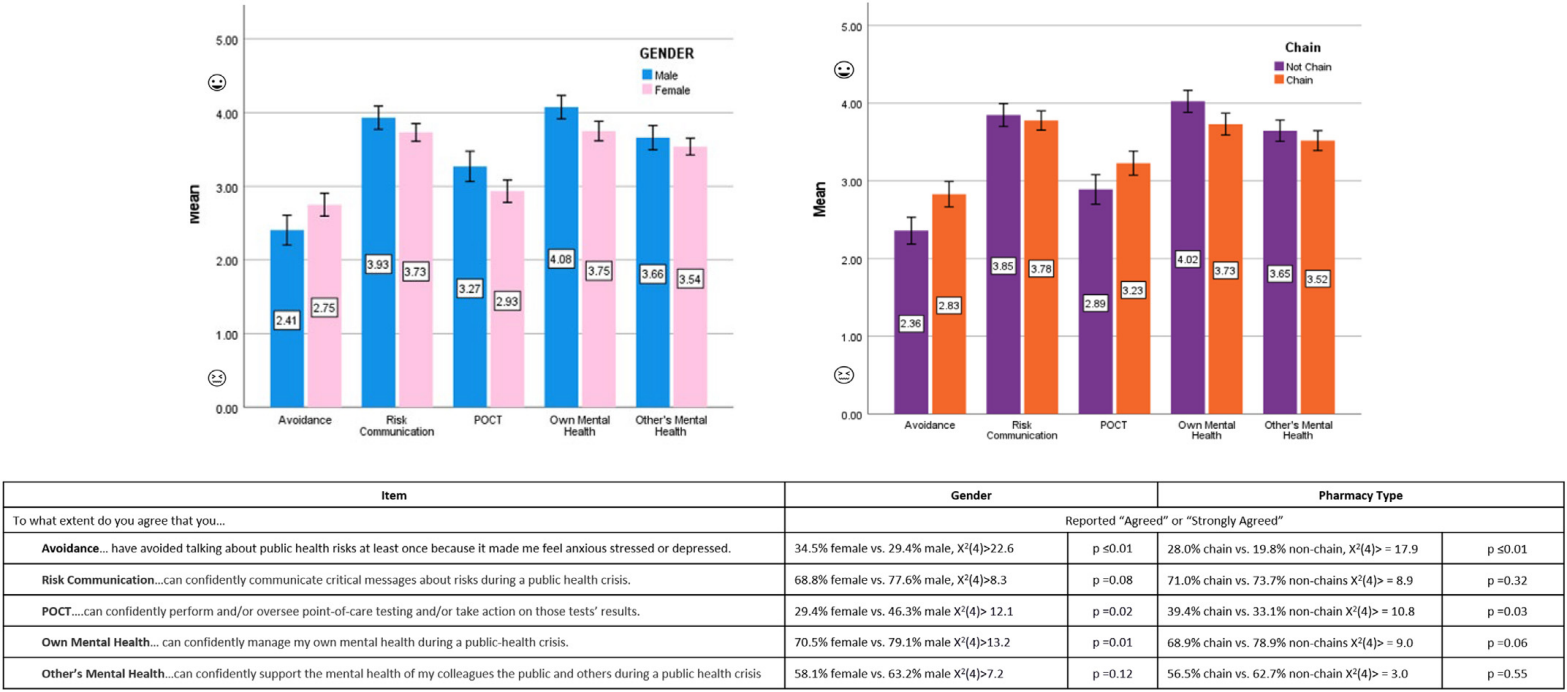
Community pharmacists' reported confidence by gender and practice site during the COVID-19 pandemic.

Descriptive statistics were computed, and survey data were analyzed in SPSS. If a respondent gave a range on an item asking for a single continuous number (e.g., weekly script count), then the average of the range was entered. Likert data were converted to five-point ordered numerical values with the highest values depicting the highest stress (i.e., never = 1, very often = 5); reverse-scored items data were inverted (i.e., never = 5, very often = 1). Normality was checked with Kolmogorov-Smirnov and Shapiro-Wilk, and any non-normal variables were transformed for correlation analysis. Likert data and respondent demographics were analyzed using chi-square tests and ANOVA, as appropriate. Alpha for all tests was set at *p* = 0.05. Free-text comments were discussed to suggest a context for aggregate survey results, but no thematic analysis was attempted. Likert data were converted to a scale to show mean and standard deviation (see Fig. 1, Fig. 2); no tests were performed on this transformation.

## 3 Results

The survey had an excellent 90% response rate, with 361 community pharmacists responding to the survey. Respondents were mostly White (74.5%), Non-Hispanic (77.8%), women (59.6%), dispensing pharmacists (84.5%) from a mix of chain (53.5%), grocery (18.0%), and independent pharmacies (18.6%). On average, respondents were 42.6 ± 11.7 years old and dispensed 1787.6 ± 2101.7 prescriptions per week.

Survey results suggested pharmacists experienced moderate levels of stress, as negative responses to PSS-10 items ranged between 6.4% to 43.3%, respectively. Overall, pharmacists had high rates of confidence in their ability to manage the pandemic, agreeing or strongly agreeing that they could manage their own mental health (73.1%), and help others manage their mental health (59.0%). Further, pharmacists were also highly confident in their ability to communicate the risks of the pandemic (72.0%), however, 28.0% of all surveyed reported that they had avoided talking about the pandemic because it made them feel “stressed, nervous or anxious.”

Pharmacists' perceived stress varied by age, gender, and setting. Women and those working in chain community pharmacies tended to report significantly higher rates of stress when compared to men and pharmacists working in non-chain community pharmacy settings (Fig. 1). Women and chain community pharmacists were also significantly more likely to report overall that they had avoided talking about public health risks because it made them feel anxious, stressed, or depressed. However, confidence to communicate critical risk messages did not differ between men and women, or between chain and non-chain community pharmacists. (Fig. 2). Further, men had significantly more confidence in their ability to manage their own mental health during the pandemic than women, and men had more confidence than women to perform, oversee, or act on point of care testing (POCT). Chain and non-chain pharmacists were equally confident with POCT, and increased confidence in POCT was significantly associated with higher prescription volume (F (4, 348) = [12.1], *p* = 0.01), and younger age (F (4, 350) = [9.7], *p* = 0.04).

Pharmacists younger than age 50 had significantly higher stress than those 50 years old and above. Younger respondents were significantly more likely to report feeling upset or nervous (χ^2^ 4, 351) = [21.0], *p* < 0.01); being upset because something happened unexpectedly (χ^2^ 4, 352) = [13.3], p = 0.01); feeling unable to control the important things in their life (χ^2^(4, 351) = [16.1], *p* = 0.03), and less confident in their ability to handle their personal problems (F(4, 351) = [21.1], p < 0.01) and irritations (χ^2^ 4, 349) = [11.3], *p* = 0.02); or believe that things were going their way (χ^2^ 4, 352) = [11.6], p = 0.02).

There were no significant associations between gender, age, and community pharmacy workplace setting. Pharmacists' confidence in their ability to support the mental health needs of others did not differ by gender, practice setting, or age. No relationships between stress, confidence, weekly-prescription volume, and race or ethnicity were found.

## 4 Discussion

With their well-established accessibility as a backdrop, community pharmacists have maintained healthcare delivery continuity and have taken on expanded roles and responsibilities in a rapidly changing work environment. Yet, despite (or perhaps because of) being at the COVID-19 pandemic's frontline, community pharmacists have experienced unprecedented stress, leading to workplace burnout and fatigue. A 2021 study estimated that >70% of community pharmacists reported burnout and stress and the COVID-19 pandemic has likely brought forth further challenges and burnout among pharmacists.^5^^,^^9^^,^^10^

Although we found generally moderate levels of stress, our findings reinforce previous study results that show being a woman, being younger in age, or being an employee of a chain pharmacy as risk factors associated with higher rates of burnout and stress.^9^^,^^11^ Distinctly, those who were most stressed before the pandemic continued to be the most stressed as this unprecedented event unfolded. These trends may be explained for two reasons. First, pharmacists employed by chain pharmacies have reported high levels of stress, poor job satisfaction, high-workplace demands, and staffing inadequacy.^11^^,^^12^ Second, research from the past two decades has shown that overall, older workers are more likely to report workplace stress than younger workers.^13^ However, recent research has shown more mixed results, with newer literature associating older age with lower rates of workplace stress specifically among pharmacists.^11^ The reasons for these mixed results are likely due to both the way age is treated (or more often ignored) as a variable in work-related stress studies, and the assumption that workplace stressors affect age groups similarly.^13^ Increased work-stress among the young may also be a result of changing workplace environments. Over the past two decades, the U.S. has experienced a major reduction of independent pharmacies in favor of chain pharmacies.

Of notable concern, however, is this study's findings on the disparity of stress between gender.

Among the general workforce, women are more likely to express emotional exhaustion, whereas men display more depersonalization (i.e., distancing oneself psychologically from the workplace).^14^ Studies exploring gender differences in stress–among pharmacists specifically– have produced mixed results. Whereas a systematic review of U.S-based pharmacists' relationships between gender and stress found mixed results nearly a decade ago,^12^ more recent studies have found stronger connections between female-gender and stress.^11^

Consideration should be made in interpreting these results as variations in cultures and personalities can affect how individuals develop, recognize, and respond to stress. For example, while most psychological literature depicts stress as a negative state, not all distress states may be bad. As such, reports of a positive association between stress and job satisfaction among licensed pharmacists (with men slightly greater than women) could be explained because in many instances, men continue to often hold higher-level positions than women and higher-level positions can be both stressful and satisfying.^15^

Cultural and subjective norms may also influence stress-gender disparities among pharmacists. Researchers surveying married pharmacists in the U.S. explained a disparity between female pharmacists' reported time and need for family commitment as a reflection of their perceived mounting workplace stressors.^16^ Unfortunately, with the present study, data were not obtained about participants' personal/family situation (e.g., parenting/eldercare responsibilities, size of family, etc.), workload, or baseline stressors, and we were unable to examine other factors that may better explain why a gender gap related to stress exists.

There are also important practical implications regarding this study's findings in that women had less confidence and avoided talking to the public about the pandemic more often than their male colleagues. Given that women are the predominant gender representing the pharmacy workforce, a lack of confidence may prevent timely, accurate, and effective communication of health risks via a Dunning-Kruger effect (i.e., a phenomena in which those with little information report higher confidence than those with more information, and those with more information underestimate their abilities).^17^^,^^18^ In the event of another sustained public health crisis, pharmacists' confidence in educating and advising the public is essential. Future research in this area may better inform how pharmacists can build the resilience necessary to anticipate, prepare, and cope with such disasters.

There are further limitations to the current study, as it only presents a snapshot of a single point in time. Policies, procedures, and knowledge about COVID-19 rapidly changed throughout the pandemic, and the environments influencing community pharmacists' responses to this survey likely changed just as quickly. Notably, vaccines and point of care testing were not widely available at the time pharmacists were queried, which may explain why stress scores were somewhat lower than anticipated. Application of these findings to future pandemics or natural disasters should be taken cautiously; given that this was most likely the first global pandemic respondents faced, it is unlikely that pharmacists of this generation will face another pandemic with as little preparation as seen during the COVID-19 pandemic.

This study had a 90% response rate, however, the proportion represented by the sample sought (i.e., 400 respondents) to the total eligible population remains unknown. This is because no public record exists within the state of Connecticut documenting the number of pharmacists working in community pharmacies. However, a likely estimate might be made if the number of community pharmacies in the state (approximately 600) and typical staffing (2–2.5 pharmacists/pharmacy), were considered, resulting in a response close to 30% of the eligible population.

Items created from the ERC framework were not validated but were checked for content and clarity by beta testers. Although this study used a validated stress measure (PSS-10), one of the 10 items was inadvertently excluded from the disseminated survey limiting our ability to calculate a composite stress score. Normally, a score is obtained by summing across all items, with higher scores indicative of higher levels of perceived stress. Without all ten items, a summative score could not be calculated, and therefore correlations were calculated between variables and each PSS-10 item, rather than any composite score. However, the PSS-10 has shown good internal consistency among various populations and is widely used as a valid and reliable indicator of stress.

Similarly, correlations between pharmacists' demographic characteristics and reported rates of stress and confidence were limited by which demographics were chosen for collection (e.g., average number of hours worked per week). Another limitation is the extent to which the sample of Connecticut pharmacists were representative of the larger population of Connecticut pharmacists and the nation. Connecticut ranks 29th in racial/ethnic diversity among other states. Regardless of these limitations, this study captured factors related to stress and confidence among a representative sample of community pharmacists at the height of the COVID-19 pandemic and provides novel data that can inform future work about how pharmacists cope and can build resilience to prepare society for the next public health emergency.

## 5 Conclusion

Overall, the COVID-19 pandemic seemed to exacerbate many existing issues. In addition, being a woman, being younger than 50 years old, or being employed by a chain pharmacy was associated with higher rates of stress and lower self-confidence among community pharmacists during the early months of the COVID-19 pandemic. Future research might explore the psychological and social mechanisms for relationships between pharmacists' stress, confidence, and burnout at times of public health crises, and how such factors affect public health crisis messaging by pharmacists and subsequent patient-care outcomes. In addition, this study calls attention to a critical need for exploring ways to ensure pharmacists in need of psychological and social support at times of public health crises are (1) identified, and (2) provided tailored resources and support to ensure their personal health and professional responsibilities are maximized.

## Funding

Funding for this project came from the University of Connecticut's Institute for Collaboration on Health, Intervention, and Policy (InCHIP).

## Declaration of Competing Interest

The authors declare that they have no known competing financial interests or personal relationships that could have appeared to influence the work reported in this paper.
